# Turing pattern–based design and fabrication of inflatable shape-morphing structures

**DOI:** 10.1126/sciadv.ade4381

**Published:** 2023-02-10

**Authors:** Masato Tanaka, S. Macrae Montgomery, Liang Yue, Yaochi Wei, Yuyang Song, Tsuyoshi Nomura, H. Jerry Qi

**Affiliations:** ^1^ Toyota Research Institute of North America, Toyota Motor North America, Ann Arbor, MI 48105, USA.; ^2^ The George W. Woodruff School of Mechanical Engineering, Georgia Institute of Technology, Atlanta, GA 30332, USA.; ^3^ Toyota Central R&D Laboratories Inc. , Bunkyo-ku, Tokyo 112-0004, Japan.

## Abstract

Turing patterns are self-organizing stripes or spots widely found in biological systems and nature. Although inspiring, their applications are limited. Inflatable shape-morphing structures have attracted substantial research attention. Traditional inflatable structures use isotropic materials with geometrical features to achieve shape morphing. Recently, gradient-based optimization methods have been used to design these structures. These methods assume anisotropic materials whose orientation can vary freely. However, this assumption makes fabrication a considerable challenge by methods such as additive manufacturing, which print isotropic materials. Here, we present a methodology of using Turing patterns to bridge this gap. Specifically, we use Turing patterns to convert a design with distributed anisotropic materials to a distribution with two materials, which can be fabricated by grayscale digital light processing 3D printing. This work suggests that it is possible to apply patterns in biological systems and nature to engineering composites and offers new concepts for future material design.

## Teaser

Turing patterns are used to convert inflatable structures with anisotropic material distributions into 3D printable designs.

## INTRODUCTION

**Fig. 1.**
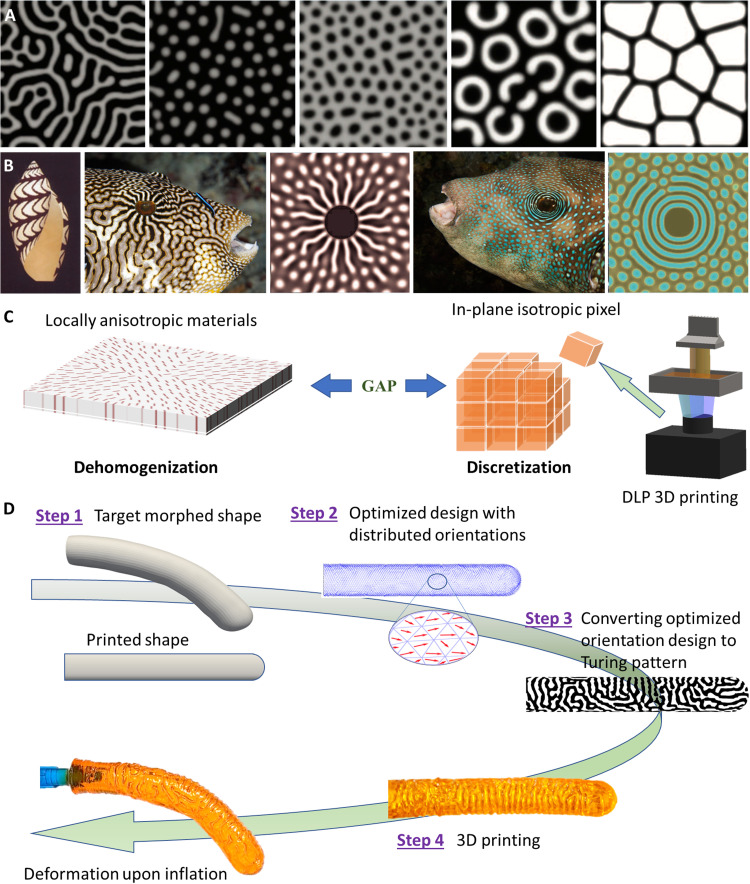
Overview of the Turing pattern–based design and fabrication of inflatable shape-morphing structures. (**A**) Turing patterns generated by the same reaction-diffusion equations with different parameter values (*4*) [reproduced with permission from the American Association for the Advancement of Science (2010)]. (**B**) Patterns seen in biological systems can be replicated by modified reaction-diffusion equations (*6*, *7*) [reproduced with permission from Springer Nature (1995) and Taylor & Francis (2006)]. Map puffer image credit: cbimages/Alamy Stock Photo. Blue-spotted puffer image credit: Aquascopic/Alamy Stock Photo. (**C**) The gap between design using orientation distribution of anisotropic materials and existing 3D printing technologies. (**D**) The integrated design framework that streamlines material orientation design optimization, converting material orientation distribution into a Turing pattern consisting of two materials (one stiff and one soft), and 3D printing the design with a Turing pattern into inflatable shape-morphing structures using a highly efficient g-DLP printing.

Turing patterns (Fig. 1, A and B) (*1*) are self-organizing patterns with stripes or spots and are widely found in biological systems and nature at multiple spatial scales, such as lizard skin (*2*), fish skins (*3*, *4*), and sand dunes (*5*). In his seminal work (*1*), Turing described how patterns can be formed autonomously from a homogeneous initial state through a process governed by reaction-diffusion equations. In Turing’s model, a pattern-forming system consists of two diffusible species, one promoting the production of both species and the other one inhibiting the production of the first. Both species diffuse and thus influence their own and the other’s concentrations, forming stripes or spots in the concentration field. The Turing pattern is regarded as a standard mathematical model and is one of the universal strategies for pattern forming in nature (*4*), not only in living biological organisms but also in nonliving systems such as patterns on sand surfaces. Figure 1A shows different Turing patterns using the same governing equations but different parameter values (*4*). Furthermore, by modifying the reaction-diffusion equations, patterns seen in biological systems can be replicated (Fig. 1B) (*6*, *7*). Inspired by the vast versatile Turing patterns that are formed autonomously, it is intriguing to find their implications in engineering, such as additive manufacturing (AM) and material design. However, applying Turing pattern to engineering problems has been very limited. Recently, Petrovic *et al.*(*8*) used Turing patterns to reconstruct orientation-controlled line and space patterns with thermally optimized vector fields. On the basis of this idea, Turing patterns have been used to inhomogenize microstructures on global structures. Dede *et al.* (*9*) designed fluidic microchannels with Turing patterns, and Zhou *et al.*(*10*) extended them into microchannel reactor designs. Nomura *et al.* (*11*) have also designed steer pathways for continuous fiber composites by optimizing the local orientation of the fiber direction. Following these studies, Ichihara and Ueda (*12*) applied the Turing pattern mechanism for path planning in fused filament fabrication three-dimensional (3D) printing of short fiber reinforced composites to enhance mechanical properties and have achieved promising results. Although these early works are promising, using Turing patterns for material design has not been explored.

Inflatable shape-morphing structures that can easily change to complicated shapes with a simple pressure input have attracted substantial research attention in science and engineering to realize complex functions in a variety of fields, including soft robots (*13*–*16*), medical equipment (*17*), and wearable assistive devices (*18*). Traditional inflatable structures are designed and manufactured with isotropic materials such that shape morphing is controlled by their initial geometrical features (*19*), stitching or sealing along curvilinear paths (*20*–*22*), Kirigami pattern (*23*), or using multiple materials through complicated fabrication (*24*–*26*). Some recent works developed shape-morphing surface-based structures through changing the local curvature of the surface by controlling in-plane anisotropic deformation via, e.g., elastic bilayers (*27*), temperature-responsive hydrogels (*28*), network of tubular air channels (*29*), or liquid crystal elastomer with locally controlled mesogen alignment (*30*). These approaches require either local geometrical features (such as local grooves) or active materials with anisotropic actuations, which also increase the fabrication complexity and limit the achievable design space. The design of inflatable shape-morphing structures thus often relies on a designer’s experience and knowledge combined with trial-and-error manufacturing to arrive at a desired final shape when inflated. Machine learning–based methods have been used to design material distributions to achieve targeted deformed shapes (*31*–*34*). However, most of the designs are limited to 1D or 2D. Recently, numerical shape and orientation optimization using gradient-based methods (*19*, *28*, *30*) involving nonlinear physical simulations has been used to determine the design parameters. In these methods, it is assumed that the material is anisotropic, and the orientation of anisotropy can vary almost freely within the structure. However, this assumption makes fabrication of these structures a major challenge. For example, Aharoni *et al.* (*30*) used photomasks with designed patterns to align the mesogens of liquid crystal elastomers. However, for each design, a photomask should be made first. In addition, such an approach may have challenges when applied to nonflat surfaces.

AM or 3D printing demonstrates considerable potential to construct optimized structures derived from computational design. Compared with traditional subtractive manufacturing processes, 3D printing can place materials in 3D pixels (voxels) to fabricate a wide range of structures with rich geometries. Recently, programmed 3D shape or 4D printing is emerging as a new paradigm in AM and receiving increasing attention because it provides the as-printed structures with the ability to transform into new shapes or to alternate functions or stiffness over time according to environmental stimuli such as air pressure, heat, humidity, electric current, or light (*35*–*37*). Therefore, 3D printing can be ideal for fabricating inflatable shape-morphing structures. However, it is challenging to achieve 3D printed anisotropic materials whose orientation of anisotropy can vary freely. Extrusion-based AM methods, such as direct-ink write, can achieve anisotropic properties (*36*, *38*), but it is challenging to vary the anisotropy freely from voxel to voxel. Digital light processing (DLP) technology is a high-speed, high-resolution method. In this technology, an ultraviolet (UV) light pattern is irradiated toward a transparent membrane window at the bottom of a vat of photocurable polymer resin (Fig. 1B, right). The light pattern causes a thin layer of the liquid resin between the transparent window and a movable build plate to solidify rapidly. The build plate is then moved upward, allowing the fresh liquid resin to refill. Then, a new image is projected into the resin vat. This process is repeated until the entire part volume has been formed. However, in this approach, the material is largely isotropic in the build plane. Although a magnetic field can be used to align short fibers coated with magnetic nanoparticles to create locally controlled anisotropy (*39*, *40*), achieving voxel-level control of anisotropy would be extremely challenging, as it requires frequently rotating the magnetic field to align the fibers toward the anisotropic orientation direction. Recently, a grayscale DLP (g-DLP) was developed that allows printing materials with markedly different mechanical properties within a single layer (*41*). Thus, it is possible to make neighboring voxels have a marked difference in mechanical properties, but each voxel is still isotropic. It is therefore still a large gap between the anisotropic material–based design and 3D printing (Fig. 1C).

Here, we present a methodology of using Turing patterns to bridge this gap. Specifically, we use the Turing pattern to convert a design with continuous anisotropic material distributions to a binary distribution with two materials, one stiff and one soft. This is achieved by replacing the anisotropic diffusion tensors with the material orientation tensors in the reaction-diffusion equations, and the obtained Turing pattern can be used for 3D printing. We develop an integrated framework that streamlines material orientation design optimization, converting material orientation distribution into a Turing pattern consisting of two materials and 3D printing the design with the Turing pattern into an inflatable shape-morphing structure using a highly efficient single-vat g-DLP (Fig. 1D) (*41*). We demonstrate this by designing inflatable shape-morphing tubular structures, which can be used for soft robotic fingers. This research represents a practical method for using the Turing pattern for material design in structure and functional applications. It also suggests that it is possible to apply patterns in nature and biological systems to engineering composites and may offer new concepts for future material design.

## RESULTS

### Overview of the integrated design and manufacturing approach

Figure 1D shows an overview of our integrated framework of the streamlined digital design and manufacturing of shape-morphing inflatable structures. In step 1, we set the distance between two edges as an objective function to be minimized for a target shape. We assume that the material is anisotropic and its orientation can be locally adjusted at each material point. In step 2, we optimize the distribution of material orientations using a gradient-based optimization method incorporated with nonlinear shell finite elements to morph the structures into the desired shape upon inflation. This results in the targeted shape. In step 3, the anisotropic reaction-diffusion equations are used to generate the Turing pattern consisting of two materials. The anisotropic diffusion coefficients in the reaction-diffusion equations are replaced by the orientation tensors derived from the orientation optimization step. In step 4, the generated binarized Turing pattern is passed as a computer-aided design file for g-DLP 3D printing. In our g-DLP 3D printing system, the formulated UV curable resin can provide a variable stiffness from stretchable elastomer to glassy polymer that can be modulated by the light intensity (see the “Materials for DLP printing” section and section S1). Last, we obtain the shape-morphing inflatable structures that can morph into desired shapes with a simple air pressure.

### Optimization design of material orientation distribution

In the orientation optimization step, we consider the structure as a thin shell. A transversely isotropic model is used to represent that the material has only one preferred direction (in the shell plane). The following six components (*a*_11_, *a*_22_, *a*_33_, *a*_12_, *a*_23_, and *a*_13_) of a symmetric orientation tensor *a* are regarded as design variables

$$[a]=\left[\begin{array}{ccc} a_{11} & a_{12} & a_{13}\\ a_{12} & a_{22} & a_{23}\\ a_{13} & a_{23} & a_{33} \end{array}\right]$$ (1) (1)

These components are not independent and subject to the first, second, and third tensor invariant conditions *I*_1_, *I*_2_, and *I*_3_ as follows

$$I_{1}=tr(a)=a_{11}+a_{22}+a_{33}=1$$ (2) (2)

$$I_{2}=\left|\begin{array}{cc} a_{22} & a_{23}\\ a_{23} & a_{33} \end{array}\right|+\left|\begin{array}{cc} a_{11} & a_{12}\\ a_{12} & a_{22} \end{array}\right|+\left|\begin{array}{cc} a_{11} & a_{13}\\ a_{13} & a_{33} \end{array}\right|$$ (3) (3)

$$I_{3}=\det(a)=0$$ (4) (4)

The second and third invariant constraints can be further realized by (*42*)

$$a_{ij}^{2}=a_{ii}a_{jj}\;\mathrm{for}\;(i,j)=\{(1,2),(1,3),(2,3)\}$$ (5)

The above orientation tensor is then used to rotate the elastic tensor **C**^t^ of the transversely isotropic material, which is defined by assuming a unidirectional fiber-reinforced composite with Young’s modulus *E*_f_ = 122 MPa and *E*_m_ = 0.7 MPa, Poison’s ratio ν_f_ = 0.49 and ν_m_ = 0.49, volume fraction *V*_f_ = 0.5 and *V*_m_ = 0.5, respectively (see the “Models” section and section S2 for details).

In the optimization process, we assume that only the material orientation tensor (Eq. 1) is adjusted at individual material points in the structure and the elastic tensor **C**^t^ is constant. To reduce the computational load, we further assume that the material orientations are designed element-wise in the finite element method (FEM) simulations. The overall optimization can be summarized as follows

$$\overset{a_{ij}(x)}{\mathrm{Minimize}}J(u)$$ (6a)

$$\text{Subject to}\;a_{ij}(x)\in [\delta_{ij}-1,1]$$ (6b) (6b)

$$g_{1}:=a_{11}+a_{22}+a_{33}-1=0$$ (6c) (6c)

$$g_{2}:=a_{ij}^{2}-a_{ii}a_{jj}=0\;\mathrm{for}\;(i,j)=\{(1,2),(1,3),(2,3)\}$$ (6d)

where *J* is the objective function, **x** is the position vector in a fixed design domain, and **u** is the displacement field obtained by FEM simulation. To avoid the numerical instability during optimization that often occur in the angular or vector representations due to the cyclic behavior of trigonometric functions (*42*), we relax the first tensor invariant constraint condition (Eq. 6c), i.e., *I*_1_ is allowed to be less than one, as *g*_1_ ≤ 0. More technical issues regarding orientation optimization are discussed in detail in (*10*, *42*). We simulate the inflation of thin-shell structures using the geometrically nonlinear FEM (see the “Models” section and section S3 for details). The total Lagrangian formulation is used to allow for large displacements and finite rotations. Internal pressure is modeled as a follower force that always acts in the normal direction of the surface with a uniform magnitude (*43*).

### Conversion of material orientation distribution to Turing pattern

As schematically shown in Fig. 1C, there is a gap between material orientation design and 3D printing, as each pixel in 3D printing is isotropic. We use the Turing pattern to create a binarized hard-soft material distribution that is equivalent to the anisotropic material field in the thin wall of inflatable structures. Here, the binarized Turing pattern is generated by solving the anisotropic reaction-diffusion equations (*9*), which have two unknowns, *U* and *V*, that can be interpreted as two interacting chemical substances (in our case, *U* and *V* represent stiff and soft materials and each volume fraction is 0.5, respectively). The anisotropic reaction-diffusion equations are given by

$$\frac{\partial U}{\partial t}=\nabla \cdot (D_{u}\nabla U)+R_{u}(U,V)$$ (7a)

$$\frac{\partial V}{\partial t}=\nabla \cdot (D_{v}\nabla V)+R_{v}(U,V)$$ (7b)

where ∇ denotes gradient operator, *R*_u_(*U*,*V*) and *R*_v_(*U*,*V*) are interactive reaction terms, **D**_**u**_ and **D**_**v**_ are anisotropic diffusion coefficients. The reaction terms *R*_u_(*U*,*V*) and *R*_v_(*U*,*V*) are defined as

$$R_{u}(U,V)=a_{u}U+b_{u}V+c_{u}-d_{u}U$$ (8a)

$$R_{v}(U,V)=a_{v}U+b_{v}V+c_{v}-d_{v}V$$ (8b)

where *a*_u_, *b*_u_, *c*_u_, *d*_u_, *a*_v_, *b*_v_, *c*_v_, and *d*_v_ are constants. The diffusion coefficients **D**_**u**_ and **D**_**v**_ are written in an anisotropic form with a unit direction vector **n** as

$$D_{u}=(L_{u}-W_{u})n⊗n+W_{u}I$$ (9a) (9a)

$$D_{v}=(L_{v}-W_{v})n⊗n+W_{v}I$$ (9b) (9b)

where **I** is the second-order identity tensor, ⊗ represents dyadic product operator, *L*_u_ an*d L*_v_ are the diffusion coefficients in the **n** direction for **D**_**u**_ and **D**_**v**_, respectively, and *W*_u_ and *W*_v_ are the diffusion coefficients in the direction perpendicular to **n**. *L*_u_, *L*_v_, *W*_u_, and *W*_v_ can be designed to control the Turing pattern, such as filling space. Details on obtaining *L*_u_, *L*_v_, *W*_u_, and *W*_v_ can be found in the “Models” section and section S4.

Note that both **n**⊗**n** and **a** (in Eq. 1) are symmetric orientation tensors. We thus connect the material orientation field with the Turing pattern using

n⊗n≡a (10) (10)

Here, we have the connection between the material orientation field and direction of Turing pattern. Namely, the material orientation tensor **n**⊗**n** at every point defines the anisotropic diffusion coefficients **D**_**u**_ and **D**_**v**_ at the corresponding point. By replacing **n**⊗**n** with **a**, solving Eqs. 7a and 7b produces a Turing pattern that captures the optimized material orientation field defined by **a**. Thus, the continuous material orientation field is converted to binarized stripe pattern using dehomogenization technique, which is a postprocessing procedure that aims to restore stiffness and preferred direction of the corresponding anisotropic material. The final properties of the binarized stripe pattern are determined by the law of mixtures as described in section S2.

**Fig. 2.**
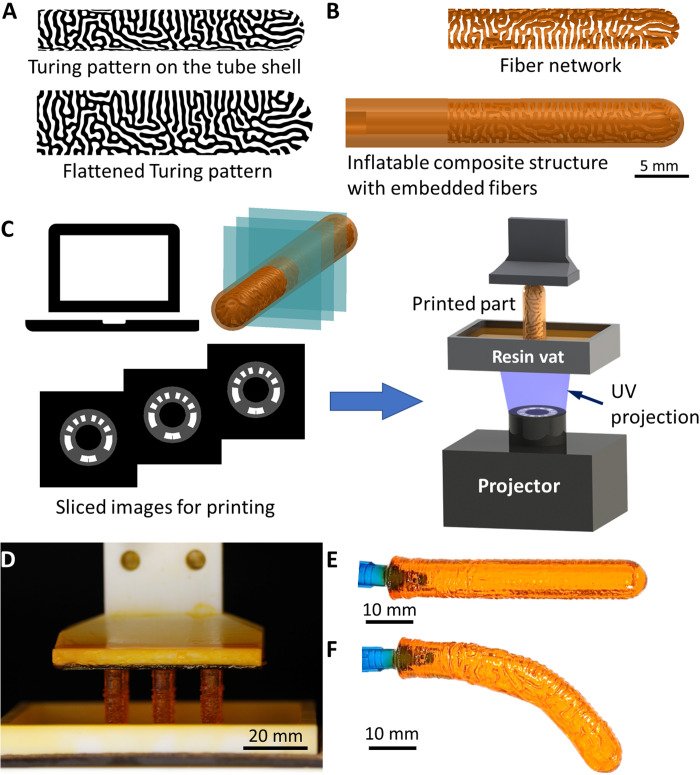
Fabrication of Turing pattern inflatable structures using g-DLP 3D printing. (**A**) Converted Turing pattern on a tube shell and the flattened image of the same pattern. (**B**) Designed Turing pattern in tubular form. The hard material serves as fiber embedded in the soft material matrix. (**C**) Computer-aided design data with the Turing pattern are sliced with grayscale patterns in individual layers for g-DLP printing. (**D**) Six tubes are simultaneously printed. (**E**) Printed tube in undeformed state. (**F**) Printed tube upon inflation.

The reaction-diffusion equation is solved in undeformed shape using multiphysics solver COMSOL (COMSOL Inc., Burlington, MA, USA) until a stable pattern is obtained. Figure 2A shows a converted Turing pattern on a tube shell. The same pattern is flattened for a better view. Here, dark regions represent the hard material and are called fibers thereafter. As it can be seen, there is no obvious anisotropy in the pattern. We attempt to quantify the similarity between the optimized anisotropic pattern and the solved Turing pattern in the fig. S3.

### Grayscale DLP 3D printing

The complex Turing patterns generated are then printed by g-DLP 3D printing, which can efficiently fabricate multimaterial structures using a single vat of resin (*41*). A special ink is designed to allow high stretchability at low light intensity and high stiffness at high light intensity (see the “Materials for DLP printing” section and section S6 for stress-strain behaviors of these materials). Because the same ink is used for printing, there is no interface between soft and stiff material. This combination of excellent interfacial strength, tunable material properties, and high stretchability makes this material system ideal for the fabrication of inflatable composite structures. Because the obtained Turing pattern is based on shell elements, it is a surface pattern. For 3D printing, it is extruded in the radial direction to form a fiber pattern network within a hollow, uniform cylinder, which has the inner and outer diameters of 5.75 and 7.85 mm, respectively. The extruded pattern (or the fiber network) is then embedded in the middle of a thick layer of matrix material with a thickness of 2.65 mm, as shown in Fig. 2B. The tube geometry with embedded Turing pattern fiber network is then sliced. The fiber regions represent the stiffer material and thus are bright; the matrix regions are soft and thus are gray (Fig. 2C). The slices images with different bright and gray regions are then used for DLP printing. Because of the small diameters of the tubes, six tubes can be printed simultaneously (Fig. 2D). More tubes can be printed simultaneously if necessary. Figure 2E shows a printed tube. Overall, the Turing pattern is visible because of the difference in the refractive index of the stiff and soft materials, but the surface of the tube is smooth. As the tube is inflated, the tube bends as designed. Because the soft material has more deformation, a surface texture with the Turing pattern is obtained. These surface textures have the advantage for grabbing soft objects, as discussed below.

### Examples of implementation

**Fig. 3.**
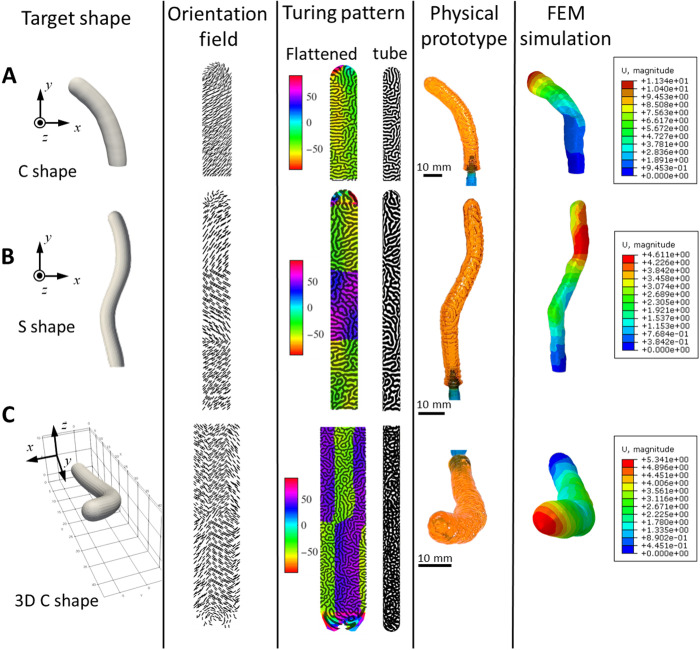
Design of inflated shapes. Different target shapes, corresponding Turing patterns, physical prototypes, and FEM simulations for (**A**) C shape, (**B**) S shape, and (**C**) 3D C shape.

By using the above-discussed design and 3D printing process, we present three different designs to demonstrate the capability of using Turing patterns for inflatable structures. Figure 3 shows designs for three target-inflated shapes: C shape, S shape, and 3D C shape, all of which are inflated from simple straight tubes. The C shape involves a simple bending upon inflation, the S shape involves two localized bending toward two different directions, and the 3D C shape involves bending and twisting. The total length of the inflation samples are 46.0, 66.0, and 65.4 mm for the C-shape design, S-shape design, and the 3D C-shape design, respectively. For g-DLP 3D printing, the stiff material is printed using 100% of light intensity (7.4 mW/cm^2^) or 0% grayscale. For simplicity, this material is named G0 material. Likewise, the light with 60% brightness (or 40% grayscale) is used to fabricate the matrix material (named G40). As shown in the Supplementary Materials, the G0 material is in a glassy state, and the G40 matrix is in a rubbery state at room temperature; the modulus of the G0 material is 60× higher than that of the G40 material.

The second column in Fig. 3 shows the anisotropic orientation fields obtained from the optimization procedure for each design. Each line represents the local orientation vector for a shell element, and the lengths of all these vectors are equal. The orientation vectors are then passed into the anisotropic reaction-diffusion equation used to generate the Turing patterns as described in the “Conversion of material orientation distribution to Turing pattern” section. This procedure generates the final fiber pattern for the targeted design as seen in the third column in Fig. 3. For C-shape and S-shape designs, because their deformations are in the plane, only half tubes are designed. For the 3D C-shape design, the Turing pattern on the entire tube is designed, so it is wider. Although the pattern in Fig. 2A does not show apparent anisotropy, we overlay color maps representing the angle of the local orientation vector with respect to the positive *y* axis on the flattened patterns to gain insight. In Fig. 3A, the yellow region represents orientation vectors closer to −90°, which leads to a horizontal-dominated Turing pattern in that area. Outside this region, vectors in the green area have a more vertical orientation, which leads to a corresponding shift in the dominant direction of the fibers. This example illustrates how the optimized orientation vectors affect the layout of the fibers through the evolution of the reaction-diffusion equations. In Fig. 3 (B and C), there are sharp transitions in the color, which signifies that the optimization procedure converged on a design with a discontinuity in the orientation field. However, the continuous Turing pattern indicates that this discontinuity has no negative impact on the fiber pattern. The reaction-diffusion procedure easily smooths over this sharp transition.

As we inflated these tubes, they deformed to the targeted shape (the fourth column) with pattern textures clearly visible on the surface, verifying that the orientation vectors had the desired effect on the reaction-diffusion procedure. To confirm that the obtained shape changes are due to the patterns, we conducted FEM simulations (for model details, see the “Models” section and section S7). The simulation results confirm that the heterogeneous design of the Turing pattern provides the driving force for the tubes to deform into the desired shapes. Surface textures such as small bulges due to the soft material are also captured by the simulations. Movie S1 shows the inflation of each tube and their comparison with FEM simulations. Quantitative comparisons of the actuated shapes and an example of more intricate optimized deformation are provided in the sections S8 and S9, respectively.

### Applications of the designed inflatable structures

As discussed above, the printed structures have a simple tubular shape but can be inflated into different configurations based on the embedded Turing patterns. Here, we demonstrate the potential applications of the inflatable shape-morphing structures.

**Fig. 4.**
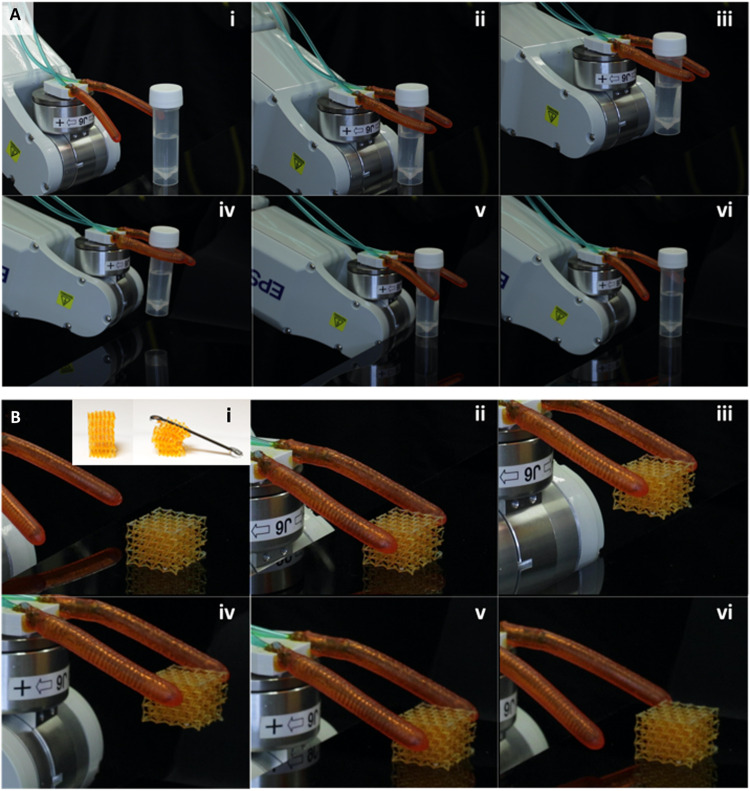
Demonstration of robotic fingers. (**A**) Pick and place of cube-shaped object. (**B**) Pick and place of a soft light-weight lattice without deforming it.

We show that the C-shape inflatable structures can be used as robotic fingers to grab objects like a pair of chopsticks. Here, we used a pair of inflatable composites and installed them on a light-weight connector fixed on the flange of an Epson C4 6-axis robot (Epson, Suwa, Nagano, Japan) as shown in Fig. 4A. The two inflatable composites have the same Turing pattern designed to bend in C shape upon inflation. For these tests, the length of the C shape is increased to 68.15 mm, and the light dose is increased to make the actuators more effective grippers. When pressure is applied to the actuators, the composite pair bend toward each other to grasp an object. When the clutch is tight, the pressure is held constant to maintain the shape of the fingers to firmly hold the object. Then, the robot moves and carries the object to a designated destination. Upon arrival, the fingers are gradually deflated to release the object. In Fig. 4A, the two fingers (each one weighs 4.02 g) could carry a research tube containing 12.8 ml of solution with cap on weighing 24.26 g in total. The textures on the surface of the soft fingers due to the Turing patterns are beneficial because they improve the gripping performance by creating extra contact area. Compared to our C-shape design (payload ratio of 3.0×), works that report the weight of the payload relative to the gripper report a maximum payload ratio of up to 7.3× (*44*) or worse: 4.5× (*45*), 3.3× (*46*), and 0.1× (*47*). Similarly, the measured value of force per total volume for our C-shape design is measured to be 94 mN at 53 kPa or 0.028 mN/mm^3^. This is within the range of 0.0069 to 0.339 mN/mm^3^ that we estimated from other works with similar soft actuators (*44*, *48*–*51*). Therefore, according to both metrics reported here, our C-shape design shares similar inflation force properties to those previously reported in the literature.

Figure 4B shows that the robotic fingers can grasp a very soft lattice while barely deforming it, demonstrating that our soft inflatable structure can handle very gentle objects, which is often a desirable property for soft grippers. The inset in Fig. 4Bi shows the lattice being crushed by a small 7.2-g wrench. The lattice has a stiffness of 2 kPa and will buckle under a mass of only 5 g. Movies S2 and S3 show the grabbing processes for the water tube and soft lattice, respectively.

## DISCUSSION

Although Turing patterns are widely seen in nature and biological systems, they have not been widely studied in engineering or, in particular, in material design and AM. In this work, we used the Turing patterns to bridge the gap between material orientation–based optimization design and 3D printing. The Turing patterns convert the orientation distribution of anisotropic materials into the binary hard-soft material patterns by coupling the material orientation with the directional tensor of anisotropic diffusivity. The inflated structures show desired shape morphing, indicating that the generated Turing patterns have successfully translated one type of material design (material orientation design) to a different one (stiff-soft material pattern design). These promising results also lead to many interesting scientific questions for future studies. First, the mechanism of this successful translation is yet to be understood. Although the obtained Turing patterns show some connection with the anisotropic orientations, it is unclear how these patterns lead to similar deformation given by the anisotropic materials. One possible reason is that anisotropic diffusion leads to a pattern with more stiff materials being placed in one direction than another, in a similar manner as the material orientation distribution. Second, some information during the Turing pattern conversion is lost. We found that for the obtained Turing pattern, the properties of stiff and soft materials different from the fiber and matrix materials in orientation distribution design can be used to obtain a similar shape morphing. On one hand, this is understandable, as the reaction-diffusion equations represent a different physics. On the other hand, this also indicates that the obtained Turing pattern represents a more general design. We found that by using different modulus ratios between the stiff and soft materials, we could obtain the similar shape morphing (see section S10). Third, it is also intriguing to determine whether we can decipher the Turing pattern to obtain the original material distribution through analyzing the pattern without conducting FEM simulations. This could have important implications for other future research. For example, can we decipher the Turing patterns seen in biological systems and investigate whether they have any connections to the functions of these systems? It should be noted that the actuated shapes in this work are relatively simple. However, this does not prevent from illustrating the use of the Turing pattern to guide the material design. In addition, we believe that more intricate shapes can be realized when the more complex designs are available. In summary, this work represents a practical framework for using the Turing patterns to guide the design of materials for structural and functional applications. The promising results indicate that the Turing pattern can serve as a biomimetic strategy for future material designs.

## MATERIALS AND METHODS

### Materials for DLP printing

The resin used in this work was a combination of urethane acrylates [EBECRYL 8413, Allnex, Inc. (North America), Alpharetta, GA, USA], isobornyl acrylate (Sigma-Aldrich, St. Louis, MO, USA), and 2-hydroxyethyl acrylate (Sigma-Aldrich) in a weight ratio of 1:3:1. Photoinitiator Irgacure 819 and photoabsorber Sudan I were added with 1 and 0.05 weight %, respectively. This formula allows the photocured material to have a high stretchability at low degree of cure (DoC) and high stiffness at high DoC. A more detailed chemical depiction is provided in the fig. S1. The mechanical behaviors of the printed materials at different light intensities were characterized using an electromechanical load frame (C41.103, MTS Systems Corporation, Eden Prairie, MN, USA). The stress-strain curves are shown in fig. S4.

### g-DLP printing and inflation tests

An in-house–built DLP printer was used in this study. The printer consists of a projector (PRO4500, Wintech Digital, Carlsbad, CA, USA) and a z-motion stage (LTS150, Thorlabs Inc., Newton, NJ, USA). The resin reservoir has an oxygen-permeable sheet to allow easy separation. During printing, we kept the exposure time constant for each layer and solely adjust the light intensity, which can be individually controlled at each pixel. The dim regions were exposed to the projection every 25 μm, but the bright regions were only exposed every 50 μm to avoid overpenetration of the light. To inflate the composite structure, a syringe nozzle (22 gauge) was inserted and glued to the prefabricated hole on the left end of the structure using the same resin used during printing as shown in Fig. 2E. For inflation tests, samples were inflated at constant pressure using a Fisnar DC200 precision valve controller (Fisnar, Germantown, WI, USA). The pressure used for the C-shape design was 50 kPa, the S shape was 50 kPa, and the 3D C shape was 90 kPa. For gripper experiments, the volume of air was manually controlled using a 50-ml syringe for each tube.

### Models

A transversely isotropic material model was used in the inverse simulation. The material constants in the elastic tensor for the model were calculated on the basis of unidirectional fiber-reinforced composites (*52*). The elastic tensor was rotated as the material orientation (fiber direction) changes (see section S2 for details). In the orientation design optimization step, this transversely isotropic material model was used in nonlinear three-node shell finite elements (see section S3 for details) in an in-house developed FEM and optimization design package (see section S3 for details). In the anisotropic reaction-diffusion model, *L*_u_, *L*_v_, *W*_u_, and *W*_v_ in Eqs. 9a and 9b can be further decomposed to control the Turing patterns. For details, see section S4. The effects of these parameters on the final pattern and deformation performance are studied in fig. S2. The commercial FEM simulation package ABAQUS\Explicit (Dassault Systemes Simulia Corp., Johnston, RI, USA) was used to simulate the inflation process of the designed structure with the Turing patterns. To model the inflation process, we used a fluid cavity interaction with mass flux and 3D shell structure simulations using reduced integration quad and tri elements (S4R and S3). A Neo-Hookean hyperelastic constitutive model was used in these simulations. For details, see section S7. The sensitivity of the actuated shape on the chosen properties is studied in fig. S7.

## Supplementary Materials

### This PDF file includes:

Sections S1 to S10 Figs. S1 to S7 Table S1 Legends for movies S1 to S3 References
