# Supplementary material for: Turing pattern–based design and fabrication of inflatable shape-morphing structures
Source: Sci Adv. 2023 Feb 10;9(6):eade4381. doi: 10.1126/sciadv.ade4381 (PMC9916983; doi:10.1126/sciadv.ade4381)
Supplement: Supplementary file 1 — Sections S1 to S10 Figs. S1 to S7 Table S1 Legends for movies S1 to S3 References [file sciadv.ade4381_sm.pdf]

Science Advances
AAAS

Supplementary Materials for

Turing pattern–based design and fabrication of inflatable shape-morphing structures

Masato Tanaka *et al.*

Corresponding author: Masato Tanaka, masato.tanaka@toyota.com; H. Jerry Qi, qih@me.gatech.edu

Sci. Adv. **9**, eade4381 (2023)
DOI: 10.1126/sciadv.ade4381

The PDF file includes:

Sections S1 to S10
Figs. S1 to S7
Table S1
Legends for movies S1 to S3
References

Other Supplementary Material for this manuscript includes the following:

Movies S1 to S3

S1. Chemical Schematic of Grayscale Polymerizable Material

The UV curable ink formula used in this work is specially designed for grayscale digital light processing 3D printing. All components consist of moiety for hydrogen bonding. Thus, at lower conversion (G40) the printed part is a soft and rubbery organogel with abundant hydrogen bonding dominating the network properties; meanwhile, at high conversion (G0) the polymerized hard block (isobornyl acrylate) turns it into a glassy thermoset dominating the network properties. The detail is illustrated in **Figure S1**.

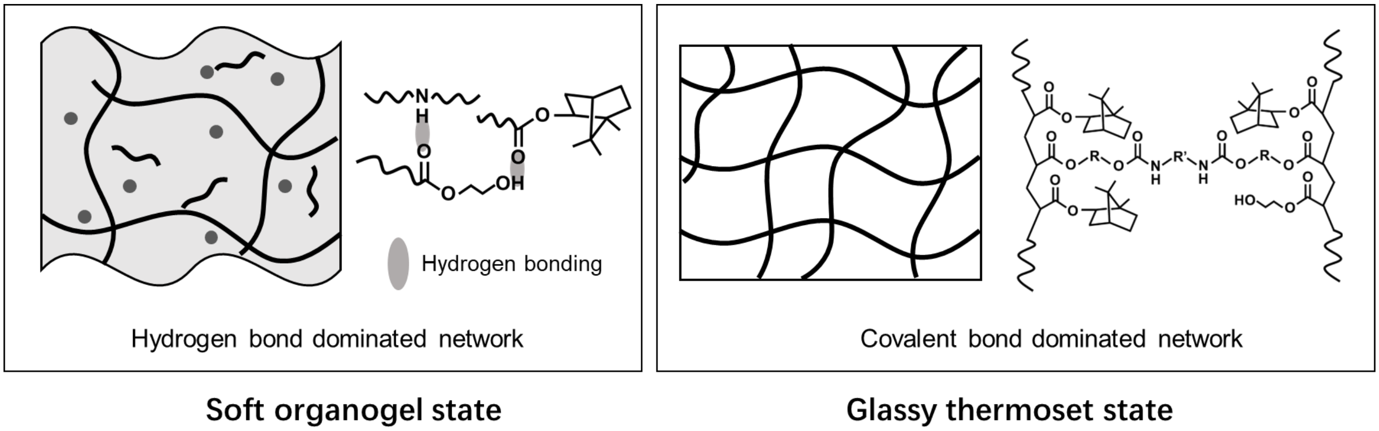

Figure S1: Depiction of the chemical makeup of the UV curable ink used to obtain orders of magnitude stiffness difference.

S2. Transversely isotropic material model

Here, the fourth-order elastic tensor of the transversely isotropic material C^t is provided in the Voigt notation as follows:

$$[\boldsymbol{C}^t] = \begin{bmatrix} C_{1111}^t & C_{1122}^t & C_{1122}^t & 0 & 0 & 0 \\ & C_{2222}^t & C_{2233}^t & 0 & 0 & 0 \\ & & C_{222}^t & 0 & 0 & 0 \\ & & & C_{2323}^t & 0 & 0 \\ & \text{symm.} & & & C_{1212}^t & 0 \\ & & & & & C_{1212}^t \end{bmatrix} \qquad (S1)$$

C_{ijkl}^t $(i, j, k, l = 1,2,3)$ is given as

$$C_{1111}^{t} = (1 - \nu_{23})\frac{E_1}{B}, \tag{S2a}$$

$$C_{2222}^{t} = (1 - \nu_{21}\nu_{12})\frac{E_2}{AB}, \tag{S2b}$$

$$C_{1122}^{t} = \nu_{12}\frac{E_2}{B}, \tag{S2c}$$

$$C_{2233}^{t} = (\nu_{23} + \nu_{21}\nu_{12})\frac{E_2}{AB}, \tag{S2d}$$

$$C_{1212}^{t} = G_{12}, \tag{S2e}$$

$$C_{2323}^{t} = (C_{2222}^{t} - C_{2233}^{t})/2, \tag{S2f}$$

$$A = 1 + \nu_{23}, \tag{S2g}$$

$$B = 1 - \nu_{23} - 2\nu_{21}\nu_{12}. \tag{S2h}$$

Here, the principal axis is assumed to be placed in the x_1-direction. In the equations above, E_1 and E_2 are the Young's moduli in the x_1-direction and other direction, respectively. ν_{12}, ν_{21}, and ν_{23} are the Poisson's ratios, G_{12} is the shear modulus, and G_{12} and ν_{21} are defined as

$$G_{12} = \frac{E_2}{2(1 + \nu_{21})}, \tag{S3a}$$

$$\nu_{21} = \nu_{12}\frac{E_2}{E_1}. \tag{S3b}$$

In this work, we assume a unidirectional fiber composite with fiber volume fraction V_f, the moduli of elasticity in the longitudinal direction E_1 and in the transverse direction E_2 are given using a general rule of mixtures as follows:

$$E_1 = V_f E_f + \left(1 - V_f\right)E_m, \tag{S4a}$$

$$E_2 = \frac{1}{\left(\frac{V_f}{E_f} + \frac{1 - V_f}{E_m}\right)}, \tag{S4b}$$

where E_f and E_m are the Young's moduli of the fibers and the matrix. For simplicity, we assume the fiber and the matrix have the same Poisson' ratio. In the inverse design simulations, we used E_f = 122MPa, E_m = 0.7MPa, and V_f = 0.5.

The above elastic tensor is defined in a local coordinate system defined by the fiber direction (or material orientation). As the composite is placed in a global coordinate, which is different from the material orientation, $\boldsymbol{C}^t$ is rotated by the orientation tensor a as follows

$$\begin{aligned} C^r_{ijkl} &= B_1(\boldsymbol{a})_{ij}(\boldsymbol{a})_{kl} + B_2\left[(\boldsymbol{a})_{ij}\delta_{kl} + (\boldsymbol{a})_{kl}\delta_{ij}\right] \\ &+B_3\left[(\boldsymbol{a})_{ik}\delta_{jl} + (\boldsymbol{a})_{il}\delta_{jk} + (\boldsymbol{a})_{jk}\delta_{il} + (\boldsymbol{a})_{jl}\delta_{ik}\right] \\ &+B_4\left(\delta_{ij}\delta_{kl}\right) + B_5\left(\delta_{ik}\delta_{jl} + \delta_{il}\delta_{jk}\right) \end{aligned} \tag{S5}$$

wherein δ_{ij} is Kronecker's delta, and the coefficients B_i are determined by $C_{ijkl}{}^t$ as

$$B_1 = C^t_{1111} + C^t_{2222} - 2C^t_{1122} - 4C^t_{1212} \tag{S6a}$$

$$B_2 = C^t_{1122} - C^t_{2233} \tag{S6b}$$

$$B_3 = C^t_{1212} + (C^t_{2233} - C^t_{2222})/2 \tag{S6c}$$

$$B_4 = C^t_{2233} \tag{S6d}$$

$$B_5 = (C^t_{2222} - C^t_{2233})/2. \tag{S6e}$$

S3. Finite element method simulations in inverse design

In the orientation optimization step, the above transversely isotropic material model is used in nonlinear 3-node shell finite elements of an in-house finite element method (FEM) program. The shell element is based on the formulation of the MITC3[53], whose key aspects rely on introduction of the assumed covariant transverse shear strain fields to alleviate the so-called shear and membrane locking phenomena. Sensitivities with respect to orientation variables are calculated with the shell finite element solutions using adjoint-based method. The optimization problem is solved with method of moving asymptotes (MMA)[54], which is a standard mathematical programming method for structural optimization.

S4. Parameter in anisotropic diffusion coefficients for controlling Turing patterns

In the anisotropic reaction-diffusion model, L_u, L_v, W_u, and W_v in Eq. 9 can be designed to control the Turing pattern. They can be further designed as follows

$$L_u = l_u^2 W_u, \quad W_u = (w_u w)^2, \quad L_v = l_v^2 W_v, \quad W_v = (w_v w)^2. \tag{12}$$

l_u, l_v are magnitudes of anisotropy parameter, w_u, w_v are parameters for pitches of U, V, and w is the magnitude of lateral diffusion. These parameters control the resulting space filling Turing pattern. In this study the following values are used: $l_u = 1$, $l_v = 1$, $w_u^2 = 0.02$, $w_v^2 = 0.5$, $w = 0.05$, $a_u = 0.08$, $b_u = -0.08$, $c_u = 0.04$, $d_u = 0.03$, $a_v = 0.1$, $b_v = 0$, $c_v = -0.15$ and $d_v = 0.08$.

The effect of changing some of these parameters is depicted in **Figure S2.** The denser fiber pattern has the effect of making a more accurate and continuous curvature while using a coarser pattern makes a more uneven curvature. This is expected as a finer pattern should have more detail and smaller tunable resolution, which should more accurately approximate the target orientation field. The most important feature of the pattern appears to be fiber connectivity. If the stiff phase is not adequately connected throughout the actuator, then the resulting pattern will not produce the local stiffness differences required to produce the desired deformations.

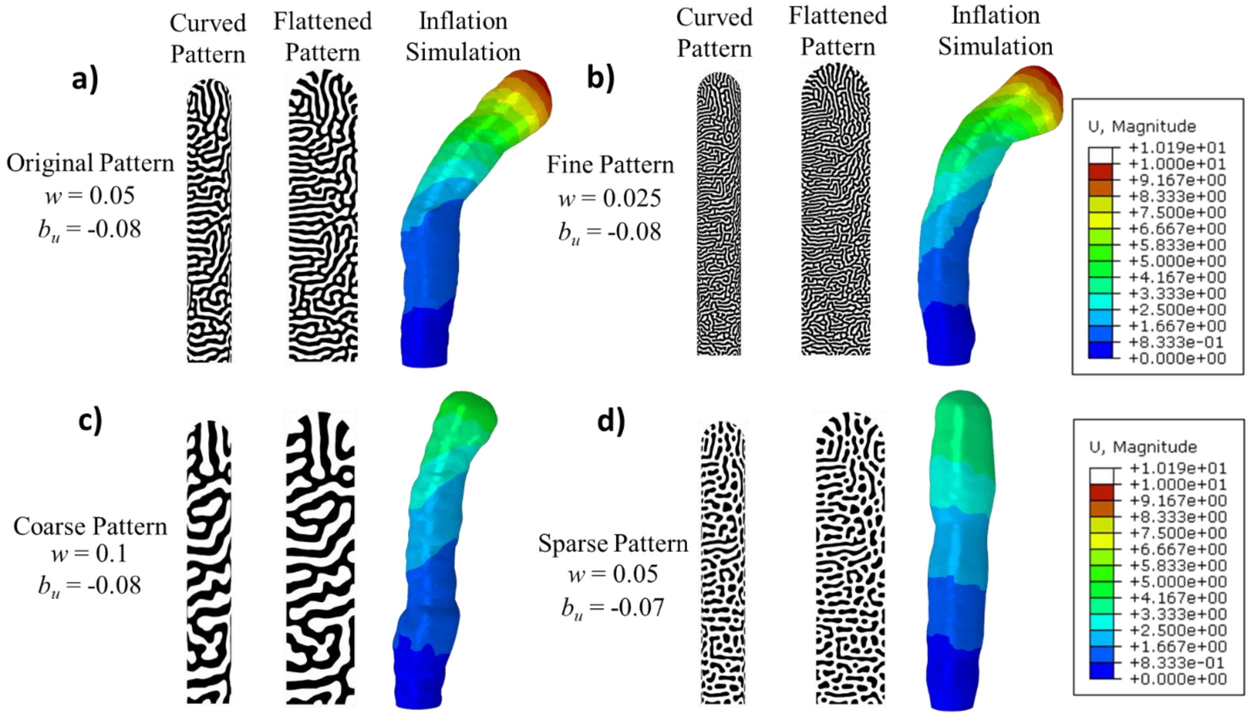

Figure S2: Effect of diffusion parameters on achieved Turing pattern. a) Original pattern presented in the main text. b) Decreasing the value of w leads to a much finer pattern. c) Increasing

w leads to a much coarser pattern. d) Leaving w unchanged and decreasing b_u has the effect of decreasing the connectivity of the fiber phase and increasing the connectivity of the matrix phase.

S5. Comparison of Optimized Orientations and Turing Pattern

In order to quantitatively compare directions between the optimized orientation field and Turing pattern, we discretized the edge lines of the Turing pattern into line elements as shown in **Figure S3a**. Then, we calculated the angles between the line elements and vectors from the optimized orientation field. **Figure S3b**, **S3c**, and **S3d** show distribution of the computed angle at each node of the line element for C-Shape, S-Shape and 3D C-shape design, respectively. In contour color of Figure S3b, S3c, and S3d, blue one indicates good agreement of their directions while yellow one means that they are perpendicular. According to those figures, although some positions show yellow colors, most of part appears in bule. Overall, the Turing pattern captures the optimized orientation field, and therefore the pattern can drive the optimized deformations. The discrepancy is because the Turing patterns are derived from reaction-diffusion equations and the effect of diffusion leads to a continuous pattern while the TO is not subject to the continuity constraint in fiber orientation.

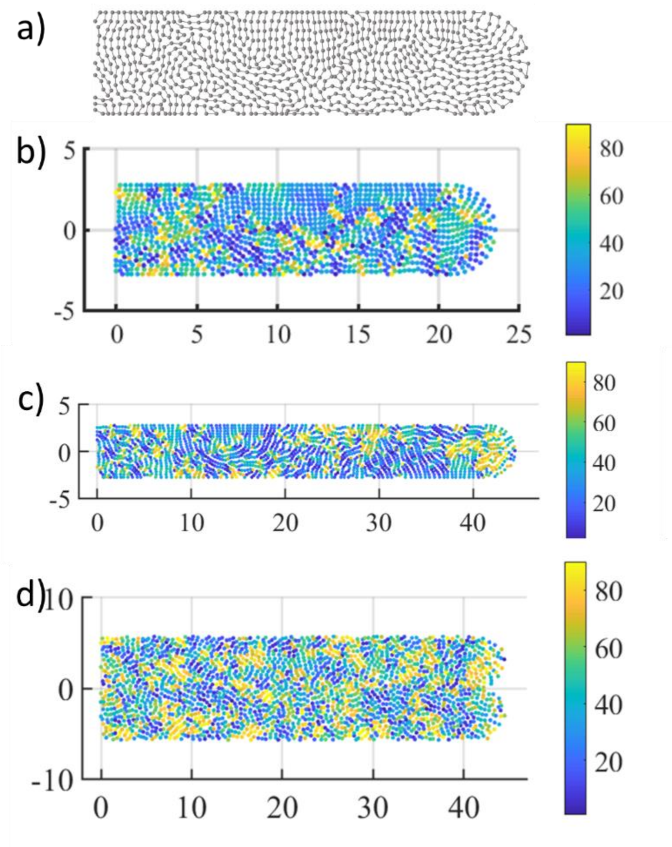

Figure S3: **Quantitative comparison of anisotropic vector fields and Turing patterns.** a) One example for discretization of edge lines of Turing pattern into line elements. Distribution of angle between the optimized orientation field and Turing pattern for b) C-Shape, c) S-Shape, d) 3D C-Shape.

S6. Stress-strain behaviors of g-DLP printed materials

The stress-strain behaviors of the g-DLP printed materials used in this work were tested by using a Criterion C41.103 Electromechanical Load Frame (MTS Systems Corporation, Eden Prairie, MN, USA). **Figure S3** shows the stress-strain curves.

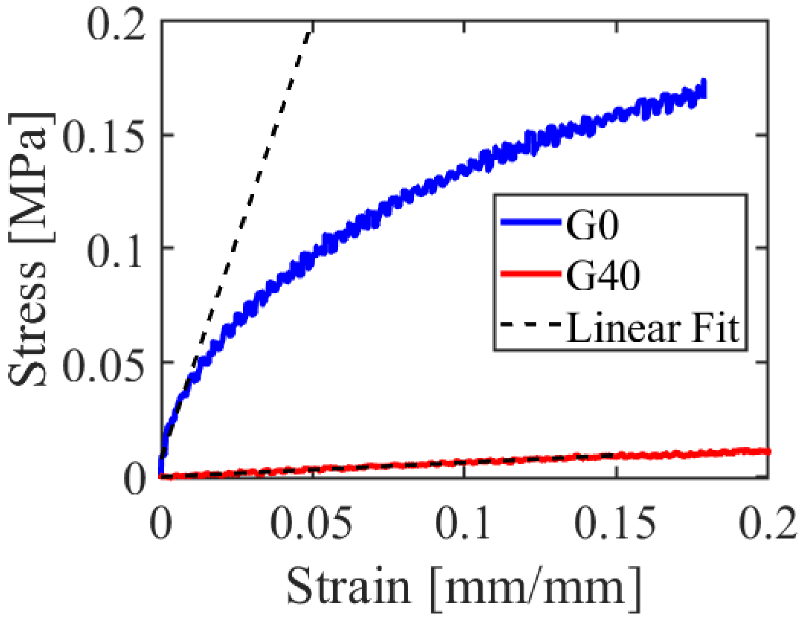

Figure S4: Stress-strain behavior of soft and stiff materials. The blue line represents the stiffer G0 material, and the red line represents the softer G40 material. Dashed lines represent linear fits for the small strain behavior.

S7. Neo-Hookean hyperelastic material model used in FEM simulations of inflated structures with Turing patterns

We used the ABAQUS\Explicit Finite Element simulation package (Dassault Systemes Simulia Corp., Johnston, RI, USA) to perform the detailed FEM simulations. We conducted 3D shell structure simulations using S4 and S3R elements using a fluid cavity interaction with mass flux to model inflation. A Neo-Hookean hyperelastic constitutive model was used in these simulations. The strain energy potential U of Neo-Hookean hyperelastic model is defined by

$$U = C_{10}(\bar{I}_1 - 3) + \frac{1}{D_1}(J - 1)^2, \tag{17}$$

where C_{10} and D_1 are material parameters, $\bar{I}_1$ is the first deviatoric strain invariant defined as $\bar{I}_1 = \bar{\lambda}_1^2 + \bar{\lambda}_2^2 + \bar{\lambda}_3^2$ with the deviatoric stretches $\bar{\lambda}_i = J^{-1/3}\lambda_i$ $(i = 1,2,3)$, and J is the total volume ratio (Jacobian). The parameters C_{10} and D_1 for stiff and soft materials used in these simulations are provided in **Table 1**. Both materials are assigned a density of 1.0×10^{-3} g/mm^3.

Table 1: Neo-Hookean constants used in detailed FEM simulation.

	C_{10} [MPa]	D_1 [MPa^{-1}]
Stiff material	0.643	0.15544
Soft material	0.01067	9.375

S8. Quantitative Inflation Comparison

Figure S5a and **S5b** show the angles that were measured for the quantitative comparison for the C-Shape and S-Shape, respectively. For the C-Shape, the bend angle is measured from the line perpendicular to the axis of the tube, which is easier to measure consistently as the tube bends compared to tracking the deflection angle of the top directly. However, since these two lines should always be perpendicular, the bend angle measurement is identical. **Figures S5c** and **S5d** show the experimental and numerical results for bend angle vs. inflation pressure for the C-Shape and S-Shape designs respectively. In both cases, the experimental results (in red) are similar to the simulated results (in black) up until a pressure around 20kPa. At this point, the simulated bend angle increases faster than the experiment show. However, by increasing the soft material from 0.064MPa to 0.096MPa, the simulated curve aligns much more closely to the experimental result. This apparent stiffness difference can be attributed to the fiber-matrix interface artificially enhancing the stiffness of the matrix material. Through light penetration and diffusion, the softer material region may be slightly stiffer than the measurement of the neat material. This difference should not have a large effect on the final shape as demonstrated in Figure S7. As indicated in our Discussion and shown in Figure S7, we did find that using different modulus ratios between the stiff and soft materials we could obtain the similar shape morphing. Therefore, the Turning pattern may represent a more general design.

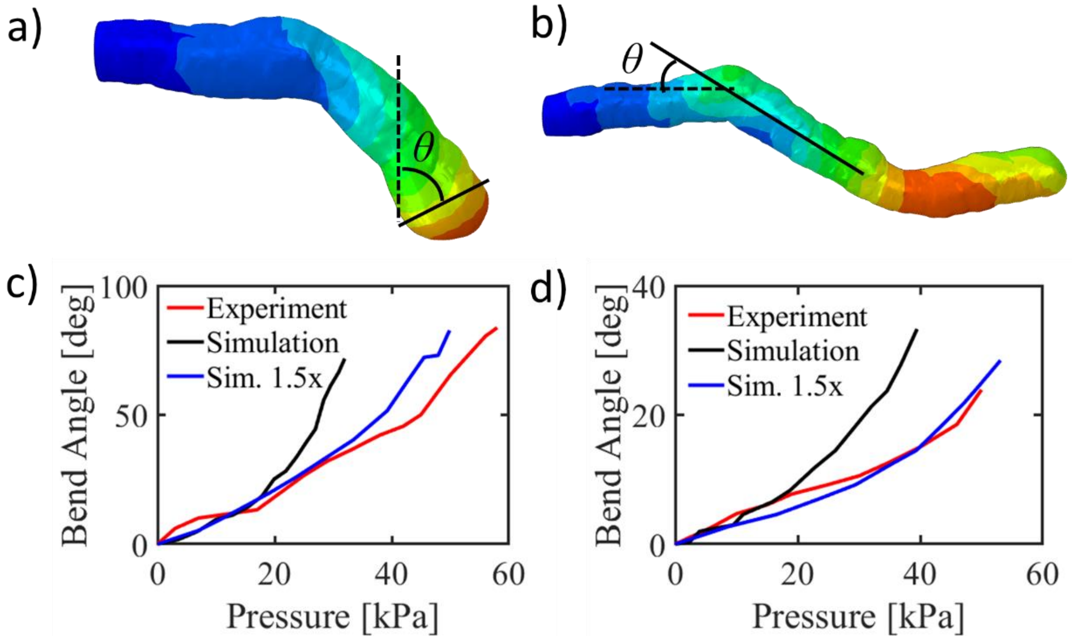

Figure S5: **Quantitative comparison of bend angle vs. inflation pressure.** a) Depiction of measured angle for the C-Shape design. b) Depiction of measured angle for the S-Shape design. c) Comparison of the bend angle at different pressures for the C-Shape experiment and simulations. d) Comparison of the bend angle at different pressures for the S-Shape experiment and simulations.

S9. Ring Structure

In this work, to perform the anisotropic material distribution optimization, an in-house topology optimization (TO) code is used. This in-house TO is limited in the complexity of the objective function definition, which prevents us from studying much more intricate deformations. The present work focuses on using the Turing pattern method to match the output of the TO design process rather than on improving the output of the TO code.

Therefore, we provide a brief example in **Figure S6** with more complicated geometry to demonstrate the broad applicability of our proposed method. **Figure S6a** shows the deformation of a ring into a saddle-like shape upon inflation from the TO simulation, and the experimental result is shown in **Figure S6b**. Although the optimized design from the TO process has limited deformation, the printed example matches well with the predicted shape.

The close match of the inflated ring structure with the target deformations from the TO design process suggests that this same method will be successful in other applications where a more complicated target deformation is available.

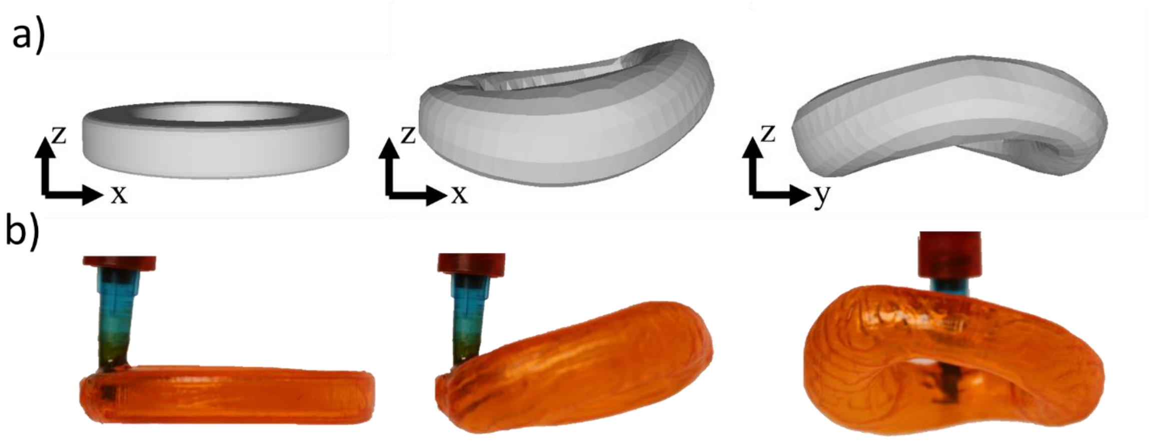

Figure S6: **Inflation optimization of ring structure.** a) Anisotropic topology optimization result of ring. b) 3D-printed ring result with Turing pattern.

S10. Influence of the properties of the soft and stiff materials

While the material property difference between the fiber and matrix is essential during the initial orientation optimization simulations, no knowledge of the properties is carried through the diffusion calculations to generate the final Turing patterns. Therefore, this section investigates the sensitivity of the final deformation on the property difference between the two material phases in the printed Turing pattern. The results of the simulations with property differences ranging from 1x to 100x are shown in **Figure S7**.

Since the required pressure changes significantly as the properties are adjusted, comparisons are made at the point of greatest similarity between simulations. It can be seen that the desired shapes do not properly form when the property difference is only 1x, and the tube inflates as one would expect for a uniform material distribution. At 10x, however, the target deformations emerge, but bulging in soft spots along the actuators prevent them from achieving a larger deformation. At 20x and beyond, the achieved shapes are all similar and close to the target shape.

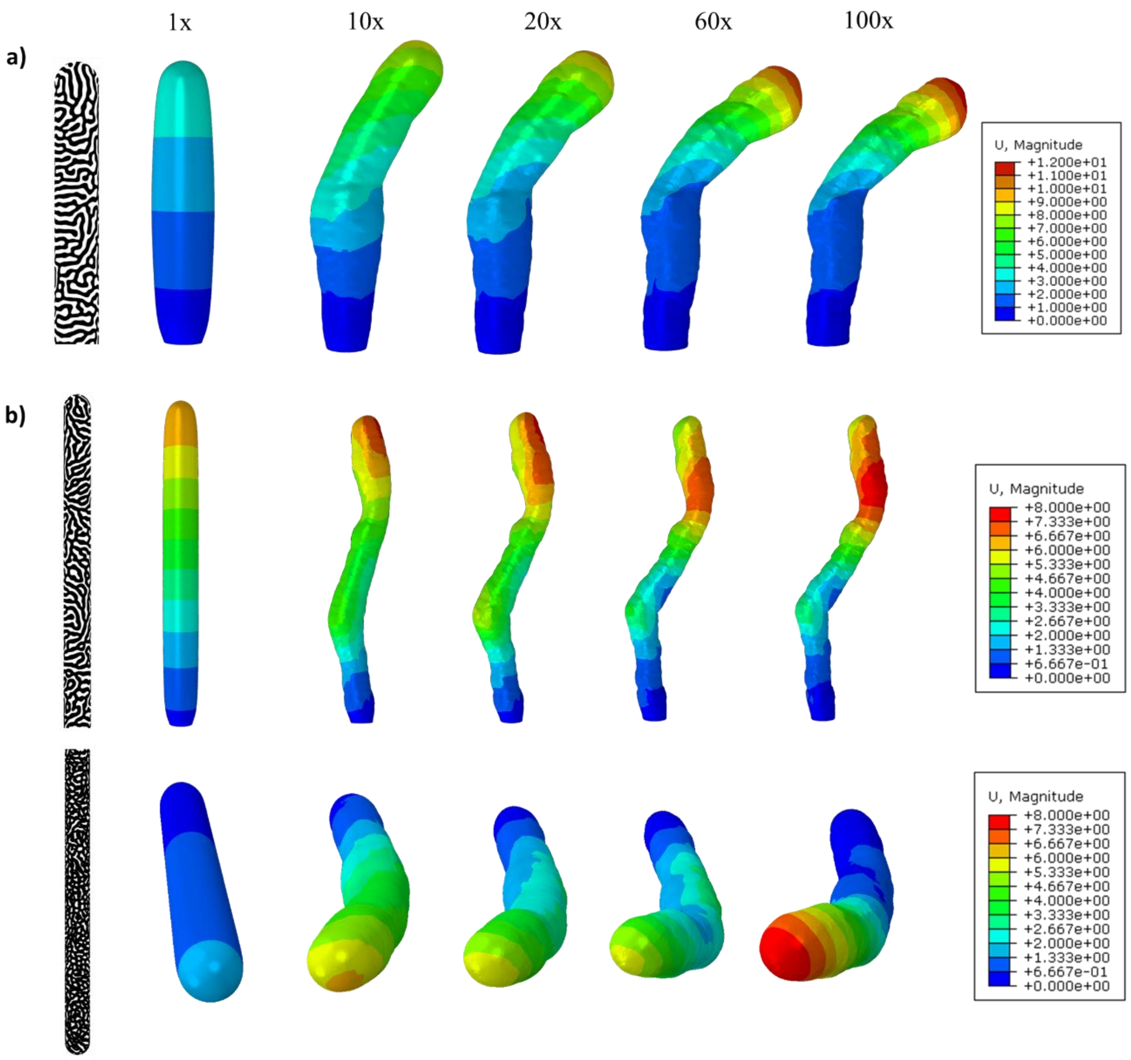

Figure S7: Inflation simulations with the ratio between the stiff and soft material ranging from 1x to 100x. Simulations for a) C-Shape, b) S-Shape, and c) 3D C-Shape.

Captions for Supplementary Videos:

Video S1: Comparison between inflation of experimental and simulated actuators.

Video S2: Pick-and-place of water tube using printed actuators.

Video S3: Pick-and-place of soft lattice using printed actuators.

REFERENCES AND NOTES

1. A. M. Turing, The chemical basis of morphogenesis. *Philos. Trans. R. Soc. Lond. B Biol. Sci.* **237**, 37–72 (1952).

2. A. Fofonjka, M. C. Milinkovitch, Reaction-diffusion in a growing 3D domain of skin scales generates a discrete cellular automaton *Nat. Commun.* **12**, 2433 (2021).

3. S. Kondo, R. Asai, A reaction-diffusion wave on the skin of the marine angelfish pomacanthus. *Nature* **376**, 765–768 (1995).

4. S. Kondo, T. Miura, Reaction-diffusion model as a framework for understanding biological pattern formation. *Science* **329**, 1616–1620 (2010).

5. P. Ball, In retrospect: The physics of sand dunes. *Nature* **457**, 1084–1085 (2009).

6. H. Meinhardt, P. Prusinkiewicz, D. R. Fowler, *The Algorithmic Beauty of Sea Shells* (The Virtual Laboratory, Springer-Verlag, 1995), xi, 204 p.

7. A. R. Sanderson, R. M. Kirby, C. R. Johnson, L. Yang, Advanced reaction-diffusion models for texture synthesis. *J. Graph. Tools* **11**, 47–71 (2006).

8. M. Petrovic, T. Nomura, T. Yamada, K. Izui, S. Nishiwaki, Thermal performance optimization in electric vehicle power trains by locally orthotropic surface layer design. *J. Mech. Des.* **140**, 111413 (2018).

9. E. M. Dede, Y. Q. Zhou, T. Nomura, Inverse design of microchannel fluid flow networks using Turing pattern dehomogenization. *Struct. Multidiscipl. Optim.* **62** 2203–2210 (2020).

10. Y. Zhou, D. J. Lohan, F. Zhou, T. Nomura, E. M. Dede, Inverse design of microreactor flow fields through anisotropic porous media optimization and dehomogenization. *Chem. Eng. J.* **435**, 134578 (2022).

11. T. Nomura, A. Spickenheuer, K. Yoshikawa, A. Kawamoto, Y. Iwano, Design and fabrication of lightweight automotive parts with continuous fiber composite using topology

optimization and tailored fiber placement. *Trans. Soc. Automot. Eng. Japan* **51**, 20197030 (2020).

12. N. Ichihara, M. Ueda, 3D-print infill generation using the biological phase field of an optimized discrete material orientation vector field. *Compos. B Eng.* **232**, 109626 (2022).

13. M. Tanaka, H. Noguchi, Structural shape optimization of hyperelastic material by discrete force method. *Theor. Appl. Mech. Japan***53**, 83–91 (2004).

14. D. Rus, M. T. Tolley, Design, fabrication and control of soft robots. *Nature* **521**, 467–475 (2015).

15. K. Suzumori, S. Endo, T. Kanda, N. Kato, H. Suzuki, A bending pneumatic rubber actuator realizing soft-bodied manta swimming robot, in *Proceedings of the 2007 IEEE International Conference on Robotics and Automation*, Rome, Italy, 10–14 April 2007.

16. S. Kriegman, S. Walker, D. Shah, M. Levin, R. Kramer-Bottiglio, J. Bongard, Automated shapeshifting for function recovery in damaged robots. arXiv:1905.09264 [cs.RO] (22 May 2019) .

17. P. W. Serruys, P. de Jaegere, F. Kiemeneij, C. Macaya, W. Rutsch, G. Heyndrickx, H. Emanuelsson, J. Marco, V. Legrand, P. Materne, J. Belardi, U. Sigwart, A. Colombo, J. J. Goy, P. van den Heuvel, J. Delcan, M. A. Morel, A comparison of balloon-expandable-stent implantation with balloon angioplasty in patients with coronary-artery disease. *N. Engl. J. Med.* **331**, 489–495 (1994).

18. P. H. Nguyen, W. L. Zhang, Design and computational modeling of fabric soft pneumatic actuators for wearable assistive devices. *Sci. Rep.* **10**, 9638 (2020)..

19. M. Skouras, B. Thomaszewski, P. Kaufmann, A. Garg, B. Bickel, E. Grinspun, M. Gross, Designing inflatable structures. *ACM Trans. Graph.* **33**, 1–10 (2014).

20. E. Siefert, E. Reyssat, J. Bico, B. Roman, Bio-inspired pneumatic shape-morphing elastomers. *Nat. Mater.* **18**, 24–28 (2019).

21. E. Siefert, E. Reyssat, J. Bico, B. Roman, Programming curvilinear paths of flat inflatables. *Proc. Natl. Acad. Sci. U.S.A.* **116**, 16692–16696 (2019).

22. E. Siefert, E. Reyssat, J. Bico, B. Roman, Programming stiff inflatable shells from planar patterned fabrics. *Soft Matter* **16**, 7898–7903 (2020).

23. L. Jin, A. E. Forte, B. Deng, A. Rafsanjani, K. Bertoldi, Kirigami-inspired inflatables with programmable shapes. *Adv. Mater.* **32**, 2001863 (2020).

24. B. Goo, C. H. Hong, K. Park, 4D printing using anisotropic thermal deformation of 3D-printed thermoplastic parts. *Mater. Des.* **188**, 108485 (2020).

25. J. H. Pikul, S. Li, H. Bai, R. T. Hanlon, I. Cohen, R. F. Shepherd, Stretchable surfaces with programmable 3D texture morphing for synthetic camouflaging skins. *Science* **358**, 210–214 (2017).

26. Q. Zhang, X. Kuang, S. Weng, L. Yue, D. J. Roach, D. Fang, H. J. Qi, Shape-memory balloon structures by pneumatic multi-material 4D printing. *Adv. Funct. Mater.* **31**, 2010872 (2021).

27. W. M. van Rees, E. Vouga, L. Mahadevan, Growth patterns for shape-shifting elastic bilayers. *Proc. Natl. Acad. Sci. U.S.A.* **114**, 11597–11602 (2017).

28. J. Panetta, F. Isvoranu, T. Chen, E. Siéfert, B. Roman, M. Pauly, Computational inverse design of surface-based inflatables. *ACM Trans. Graph.* **40**, 1–14 (2021).

29. A. Nojoomi, J. Jeon, K. Yum, 2D material programming for 3D shaping. *Nat. Commun.* **12**, 603 (2021).

30. H. Aharoni, Y. Xia, X. Zhang, R. D. Kamien, S. Yang, Universal inverse design of surfaces with thin nematic elastomer sheets. *Proc. Natl. Acad. Sci. U.S.A.* **115**, 7206–7211 (2018).

31. C. M. Hamel, D. J. Roach, K. N. Long, F. Demoly, M. L. Dunn, H. J. Qi, Machine-learning based design of active composite structures for 4D printing. *Smart Mater. Struct.* **28**, 065005 (2019).

32. S. Wu, C. M. Hamel, Q. Ze, F. Yang, H. J. Qi, R. Zhao, Evolutionary algorithm-guided voxel-encoding printing of functional hard-magnetic soft active materials. *Adv. Intell. Syst.* **2**, 2000060 (2020).

33. X. Sun, L. Yue, L. Yu, H. Shao, X. Peng, K. Zhou, F. Demoly, R. Zhao, H. J. Qi, Machine learning-evolutionary algorithm enabled design for 4D-printed active composite structures. *Adv. Funct. Mater.* **32**, 2109805 (2022).

34. A. E. Forte, P. Z. Hanakata, L. Jin, E. Zari, A. Zareei, M. C. Fernandes, L. Sumner, J. Alvarez, K. Bertoldi, Inverse design of inflatable soft membranes through machine learning. *Adv. Funct. Mater.* **32**, 2111610 (2022).

35. Q. Ge, H. J. Qi, M. L. Dunn, Active materials by four-dimension printing. *Appl. Phys. Lett.* **103**, 131901 (2013).

36. A. S. Gladman, E. A. Matsumoto, R. G. Nuzzo, L. Mahadevan, J. A. Lewis, Biomimetic 4D printing. *Nat. Mater.* **15**, 413–418 (2016).

37. X. Kuang, D. J. Roach, J. Wu, C. M. Hamel, Z. Ding, T. Wang, M. L. Dunn, H. J. Qi, Advances in 4D printing: Materials and applications. *Adv. Funct. Mater.*, **29**, 1805290 (2019).

38. S. Y. Weng, X. Kuang, Q. Zhang, C. M. Hamel, D. J. Roach, N. Hu, H. J. Qi, 4D printing of glass fiber-regulated shape shifting structures with high stiffness. *ACS Appl. Mater. Interfaces*, **13**, 12797–12804 (2021).

39. R. M. Erb, R. Libanori, N. Rothfuchs, A. R. Studart, Composites reinforced in three dimensions by using low magnetic fields. *Science* **335**, 199–204 (2012).

40. J. J. Martin, B. E. Fiore, R. M. Erb, Designing bioinspired composite reinforcement architectures via 3D magnetic printing *Nat. Commun.* **6**, 8641 2015.

41. X. Kuang, J. Wu, K. Chen, Z. Zhao, Z. Ding, F. Hu, D. Fang, H. J. Qi, Grayscale digital light processing 3D printing for highly functionally graded materials *Sci. Adv.* **5**, eaav5790 (2019).

42. T. Nomura, A. Kawamoto, T. Kondoh, E. M. Dede, J. Lee, Y. Song, N. Kikuchi, Inverse design of structure and fiber orientation by means of topology optimization with tensor field variables. *Compos. B Eng.* **176**, 107187 (2019).

43. M. J. Poldneff, I. S. Rai, J. S. Arora, Design variations of nonlinear elastic structures subjected to follower forces. *Comput. Methods Appl. Mech. Eng.* **110**, 211–219 (1993).

44. Y. Fei, J. Wang, W. Pang, A novel fabric-based versatile and stiffness-tunable soft gripper integrating soft pneumatic fingers and wrist. *Soft Robot.* **6**, 1–20 (2019).

45. D. Gonzalez, J. Garcia, R. M. Voyles, R. A. Nawrocki, B. Newell, Characterization of 3D printed pneumatic soft actuator. *Sens. Actuators A Phys.* **334**, 113337 (2022).

46. G. Zhong, Y. Hou, W. Dou, A soft pneumatic dexterous gripper with convertible grasping modes. *Int. J. Mech. Sci.* **153–154**, 445–456 (2019).

47. L. Ge, L. Dong, D. Wang, Q. Ge, G. Gu, A digital light processing 3D printer for fast and high-precision fabrication of soft pneumatic actuators. *Sens. Actuators A Phys.* **273**, 285–292 (2018).

48. G. Alici, T. Canty, R. Mutlu, W. Hu, V. Sencadas, Modeling and experimental evaluation of bending behavior of soft pneumatic actuators made of discrete actuation chambers. *Soft Robot.* **5**, 24–35 (2018).

49. D. Drotman, S. Jadhav, M. Karimi, P. de Zonia, M. T. Tolley, 3D printed soft actuators for a legged robot capable of navigating unstructured terrain. in *Proceedings of the 2017 IEEE International Conference on Robotics and Automation (ICRA)*, Singapore, 29 May–3 June 2017.

50. J. Fras, M. Macias, Y. Noh, K. Althoefer, Fluidical bending actuator designed for soft octopus robot tentacle. in Proceedings of the 2018 *IEEE International Conference on Soft Robotics (RoboSoft)*, Livorno, Italy, 24–28 April 2018.

51. Y.-F. Zhang, N. Zhang, H. Hingorani, N. Ding, D. Wang, C. Yuan, B. Zhang, G. Gu, Q. Ge, Fast-response, stiffness-tunable soft actuator by hybrid multimaterial 3D printing. *Adv. Funct. Mater.* **29**, 1806698 (2019).

52. J. Qu, M. Cherkaoui, *Fundamentals of Micromechanics of Solids* (Wiley, 2006), xiii, 386 p.

53. P.-S. Lee, K.-J. Bathe, Development of MITC isotropic triangular shell finite elements. *Comput. Struct.* **82**, 945–962 (2004).

54. K. Svanberg, The method of moving asymptotes – A new method for structural optimization. *Int. J. Numer. Methods Eng.* **24**, 359–373 (1987).
